# Foot-and-Mouth Disease Infection Dynamics in Contact-Exposed Pigs Are Determined by the Estimated Exposure Dose

**DOI:** 10.3389/fvets.2018.00167

**Published:** 2018-07-20

**Authors:** Karla I. Moreno-Torres, Barbara P. Brito, Matthew A. Branan, Luis L. Rodriguez, Amy H. Delgado, Carolina Stenfeldt, Jonathan Arzt

**Affiliations:** ^1^Foreign Animal Disease Research Unit, United States Department of Agriculture, Agricultural Research Service, Plum Island Animal Disease Center, Greenport, NY, United States; ^2^PIADC Research Participation Program, Oak Ridge Institute for Science and Education, Oak Ridge, TN, United States; ^3^United States Department of Agriculture, Monitoring and Modeling, Animal and Plant Health Inspection Service, Center for Epidemiology and Animal Health, Fort Collins, CO, United States; ^4^Department of Veterinary Population Medicine, University of Minnesota, St Paul, MN, United States

**Keywords:** FMD, foot-and-mouth disease, virus, pigs, exposure, dose, incubation, modeling

## Abstract

The quantitative relationship between the exposure dose of foot-and-mouth disease virus (FMDV) and subsequent infection dynamics has been demonstrated through controlled inoculation studies in various species. However, similar quantitation of viral doses has not been achieved during contact exposure experiments due to the intrinsic difficulty of measuring the virus quantities exchanged between animals. In the current study, novel modeling techniques were utilized to investigate FMDV infection dynamics in groups of pigs that had been contact-exposed to FMDV-infected donors shedding varying levels of virus, as well as in pigs inoculated via the intra-oropharyngeal (IOP) route. Estimated virus exposure doses were modeled and were found to be statistically significantly associated with the dynamics of FMDV RNA detection in serum and oropharyngeal fluid (OPF), and with the time to onset of clinical disease. The minimum estimated shedding quantity in OPF that defined infectiousness of donor pigs was 6.55 log_10_ genome copy numbers (GCN)/ml (95% CI 6.11, 6.98), which delineated the transition from the latent to infectious phase of disease which occurred during the incubation phase. This quantity corresponded to a minimum estimated exposure dose of 5.07 log_10_ GCN/ml (95% CI 4.25, 5.89) in contact-exposed pigs. Thus, we demonstrated that a threshold quantity of FMDV detection in donor pigs was necessary in order to achieve transmission by direct contact. The outcomes from this investigation demonstrate that variability of infection dynamics which occurs during the progression of FMD in naturally exposed pigs can be partially attributed to variations in exposure dose. Moreover, these modeling approaches for dose-quantitation may be retrospectively applied to contact-exposure experiments or field scenarios. Hence, robust information could be incorporated into models used to evaluate FMD spread and control.

## Introduction

Early detection and rapid response to a highly contagious pathogen such as foot-and-mouth disease virus (FMDV) may determine the difference between a controllable outbreak and a widespread epidemic (1, 2). The United States has been free of FMD since 1929 (3). However, approximately two-thirds of the countries that are members of the World Organization for Animal Health (OIE) report occurrence of FMD according to the OIE's World Animal Health Information Database (WAHID) (4). As part of preparedness for FMD incursions, FMD-free countries may model simulations of plausible FMD outbreaks to improve understanding of various temporo-spatial scales of spread, impact, and control (5, 6). However, outcomes of modeled approaches to any disease are limited by the extent to which basic aspects of infection have been appropriately parameterized.

Control strategies should be based on known mechanisms of pathogen transmission while also considering species-specific differences. Susceptibility to infection by distinct routes of virus exposure varies between host species (7). For example, recent experimental studies have shown that pigs are more susceptible to FMDV infection via the upper gastrointestinal tract (oropharynx) compared to the upper respiratory tract (nasopharynx) (8, 9), while cattle and sheep are more susceptible to infection via the respiratory tract (10–15). This finding is consistent with previous works demonstrating that pigs are relatively resistant to FMDV infection via exposure to aerosolized virus (16). Even though multiple routes (mechanisms) of virus entry are plausible during direct contact exposure, identification of the oropharyngeal tonsils as sites of primary and sustained viral replication in pigs suggests that transmission occurs mainly via the oral route (17). This understanding of pathogenesis of FMDV in pigs allows for the design of experimental studies which better replicate natural exposure. Such controlled experimental studies provide a foundation for our understanding of disease transmission and allow modelers to estimate disease spread and evaluate control options in a population.

Moving from experimental studies to FMD transmission models requires understanding and quantification of the factors involved in transmission. For example, the cumulative exposure dose that a contact animal receives during exposure to an infectious animal is dependent on both the amount of virus shedding from the infectious animal as well as the duration of the contact. The resultant exposure will affect the likelihood of transmission and may influence the infection dynamics in the contact animal (18, 19). However, studies estimating the dose of FMDV required to cause infection in pigs during direct contact exposure are lacking. To our knowledge, there is only one published investigation in which the exposure dose transmitted to contact pigs was estimated by quantification of FMDV RNA in nasal swabs of donor pigs that had been artificially (intradermal and subcutaneous heel-pad) inoculated with FMDV serotype O strain UKG/2001 (19). That study found that the quantity of virus shed by donors was related to transmission, and a threshold amount of virus shedding was required for transmission to occur. However, the study did not estimate the exposure dose received by contact pigs. In addition, a previous study investigating the effect of altered contact time on transmission efficiency demonstrated substantial differences between different FMDV strains (18). Hence, additional studies estimating the dose required for infection during direct contact exposure and exploring other strains of FMDV are needed.

It has been shown that altering the challenge dose of directly inoculated pigs may affect the dynamics of FMDV shedding, as well as the time to onset of viremia and clinical disease, which are critical parameters to model and predict FMDV (8, 20–22). It can thus be hypothesized that variations in the effective challenge dose during contact exposure would similarly result in varying clinical and virological progression of the disease. In particular, shedding of lower quantities of FMDV during the early phase of infection may delay the onset of infectiousness and may thereby have substantial impact on disease detection (i.e., delay the onset of clinical signs) and dissemination of infection during the early phases of an outbreak.

The aim of the current study was to model exposure doses and dose-dependency of FMD infection dynamics in pigs infected with FMDV by contact exposure to inoculated donor pigs at different stages of disease. The compiled data from different experimental IOP-inoculated and contact-exposed groups of pigs were categorized based upon similarities in infection dynamics. The effective exposure dose was estimated and compared for each of the categories with different infection dynamics. The output of this investigation may be incorporated into FMD transmission models to better predict outbreak size and severity.

## Materials and methods

### Animal experiments

The analyses presented herein are based on data derived from a series of three complementary experimental studies, defined herein as Study A, B, and C (Table 1) (8, 23, 24). These studies were originally designed for the purpose of investigating FMDV pathogenesis and transmission in pigs, with minor variation in study design across experiments. However, all of the included experiments utilized the same source virus strain under similar conditions. The virus used for all experiments was a cattle-derived strain of FMDV A_24_ Cruzeiro that had been passed once in pigs, as previously described (18). All animal experiments were performed within the BSL-3Ag containment facility at the Plum Island Animal Disease Center, NY. Experimental protocols were approved by the Institutional Animal Care and Use Committee (protocol 231-11-R).

**Table 1:** Number of IOP-inoculated and contact-exposed animals, frequency of blood and OP sample collection and clinical examination, and time points relative to initial exposure (HPI, hours post inoculation; HPE, hours post exposure).

**Study ID**	**Number of animals**				**Time points of collection**						**References**
	**IOP-inoculated**		**Contact-exposed**		**Blood**		**Oropharyngeal swabs**		**Clinical examination**		
	**Inoculation dose**		**Exposure duration**		**Sampling frequency (hours interval)**	**HPI/HPE**	**Sampling frequency (hours interval)**	**HPI/HPE**	**Evaluation frequency (hours interval)**	**HPI/HPE**	
	**10 PHID**_50_	**100 PHID**_50_	**8 h**	**24 h**							
A	2	2	–	–	24	(0–240)	24	(0–240)	24	(0–240)	(8)
B	–	2	–	–	6	(0–24)	2	(4–12)	4	(36–54)	(24)
					12	(24–96)	3	(12–24)			
					–	–	12	(24–96)			
	–	–	–	4^$^	–	12, 24 & 48	4	(0–12)	4	(0–12)	
					–	–	–	24 & 48		24 & 48	
C	–	5	–	–	–	0, 16, 24, 48 & 64	8	(0–64)	8	(0–64)	(23)
	–	–	35^#^	–	24	(0–72)^*^	8	(0–24)	8	(0–24)	
					–	–	24	(24–^*^)	24	(24–^*^)	

* *Groups were monitored until pigs develop fulminant clinical FMD or up to 21 days*.

$ *4 pigs were exposed to 4 donor pigs for 24 h starting at 36 h post inoculation of donors*.

# *7 groups of 5 pigs each were sequentially exposed to 5 infected donors for 8 h each, starting at 8 h post inoculation of donors*.

In brief, all experimental studies were comprised of pigs that were exposed through either intra-oropharyngeal (IOP) inoculation or, through direct contact exposure via cohabitation with IOP-inoculated pigs (Table 1). The IOP inoculation system is a simulated-natural inoculation system that is based on recent knowledge of FMDV pathogenesis in pigs, and that has been specifically designed to simulate natural FMDV exposure conditions (8). Amongst all 11 IOP-inoculated pigs, 9 pigs were inoculated with a dose of 100 50% pig heel infectious doses (100 PHID_50_-from studies A, B, and C) whereas 2 pigs (from study A) received a lower inoculation dose (10 PHID_50_; Table 1). The dose titration system has been previously described (18). Study A was designed to compare two novel simulated-natural inoculation methods. Study B (Table 1) was designed to identify early events in the pathogenesis of FMDV and included four additional pigs that were infected through direct contact exposure by cohabitation with two IOP-inoculated pigs. The duration of contact exposure of this study was 24 h, which corresponded to the period from 36 to 60 h post inoculation (hpi) of the donor pigs.

Study C (Table 1) was designed to determine the transition from latent to infectious phases in IOP inoculated pigs (23). A previous publication based on this study provided descriptive analyses of FMDV infection dynamics following contact-exposure, and demonstrated that transmission of FMDV occurred prior to the onset of clinical signs. The design of study C comprised seven groups of five susceptible pigs per group that were sequentially exposed to one group of five IOP-inoculated donor pigs through successive 8 h periods of co-habitation. The first group of contact pigs was introduced to the room with the IOP-inoculated donors at 8 hpi and removed from the room with the donors at 16 hpi. Subsequent contact groups were successively introduced into the room housing the donor pigs until all seven groups had been exposed, at 64 hpi. Each group of contact-exposed pigs was housed in a separate isolation room after the exposure period.

### Sample analysis

All of the studies included monitoring of FMDV infection dynamics through standardized clinical examinations and quantitation of FMDV RNA in blood and oropharyngeal (OP) swabs. Whole blood and OP swabs were collected at pre-determined time points (Table 1) and were centrifuged to extract serum and oropharyngeal fluid (OPF). FMDV quantitation was done by qRT-PCR as previously described (15, 25). qRT-PCR-generated Cycle Threshold (CT) values were converted to genome copy numbers (GCN) using the following equation [log_10_ GCN/ml = ((54.899−CT value)/3.690) + Z], where Z is 0.4 for OPF and 0.0 for serum samples, generated from analyses of a dilution series of *in-vitro* generated FMDV RNA of known quantities (25). In order to convert directly quantitated RNA values to infectious dose equivalents (expressed as 50% tissue culture infectious dose; TCID_50_), RNA was quantitated in parallel with virus titration of an FMDV A_24_ high-titer stock on LFBKαvβ6 cells as previously described (26, 27). Specifically, a 10-fold dilution series of this virus stock was subsequently analyzed by qRT-PCR (25) to generate corresponding CT values to known TCID_50_ equivalents. Thus, defining the numerical relationships between CT values to GCN and CT values to TCID_50_ equivalents, enabled the conversion from GCN to TCID_50_ equivalents (Table S1).

The progression of clinical FMD was monitored through clinical examinations during which a standardized cumulative scoring system was used to assess appearance and dissemination of characteristic (vesicular) FMD lesions (23).

### Statistical analysis

All statistical analyses were performed in R version 3.2.3 (28) in R Studio (29). Generalized Additive Mixed Models (GAMMs) using FMDV RNA dynamics in serum and OPF as dependent variables were fit using the *mgcv* package (30). Mixed effect logistic regression models modeling presence of vesicular lesions (clinical signs of FMD) as a response variable were fit using Penalized Quasi-Likelihood, implemented with the *glmmPQL* function of the *MASS* package in R.

In order to achieve comparable data sets across studies, the analyses included herein were restricted to data from ≤ 72 hpi for IOP-inoculated pigs and ≤ 120 h post exposure (hpe) for contact-exposed pigs. Overall, the analyses presented herein included a total sample size of *n* = 11 IOP-inoculated pigs and *n* = 39 contact-exposed pigs (Table 1). Merging data from these similar experiments improved sample size and allowed for more robust statistical analyses.

#### Categorizing IOP-inoculated and contact-exposed pigs based on statistical models of FMDV infection dynamics

In the analyses that follow, “groups” refer to the original grouping of the individuals within each experiment (study A, B, or C), whereas “Categories” are the newly defined clustering based on the statistical analyses performed in this study. For instance, the eight contact-exposed groups (7 from study C and 1 from B) were assigned to either Categories I, II, or III based upon differences of their infection dynamics.

GAMMs were used to estimate the appropriate categorization of the groups of pigs based on their infection dynamics. GAMMs are regression models that allow modeling a response variable as a function of smooth functions of one or more explanatory variables. These models offer a flexible approach to assess the relationships between the dependent variable and one or more explanatory variables, while accounting for random effects (31). All GAMM analyses utilized the Gaussian family with identity link, a thin plate smoothed term for time (hours), and an automatic selection of the amount of smoothing using cross-validation.

Data from IOP-inoculated pigs from the three studies were evaluated based on FMDV RNA dynamics in serum and OPF. Two distinct Generalized Additive Mixed Models (GAMMs) were fitted to the data. The levels of FMDV RNA in serum or OPF were the dependent variables, whereas time (hours) and an indicator variable for the challenge dose (low 10 PHID_50_ vs. high 100 PHID_50_) were the explanatory variables for each model. Individual animal identification was used as a random effect to account for repeated measurements on the same animal. If the groups did not differ significantly (significant differences were determined using *p* < 0.05 for between group comparisons), they were combined into a single category for further analysis.

Once the data from IOP-inoculated pigs were appropriately combined based on the levels of FMDV RNA in serum or OPF, the fitted curves and 95% confidence intervals (CI) of the GAMM analyses and the analytical lower limit of detection (LOD) for FMDV RNA as determined for the qRT-PCR detection system were used to estimate the time to FMDV RNA detection in OPF and serum for each category of animals. The analytical lower limit of detection was calculated to be 2.68 log_10_ GCN/mL for serum and 3.08 log_10_ GCN/mL for OPF due to different dilution steps during sample processing. Specifically, the time to the earliest detection of FMDV RNA for serum and OPF samples was the time at which the fitted curve reached the respective LOD.

A similar process was then used to evaluate how to categorize the IOP-inoculated pigs based on the presence of vesicular lesions (clinical signs of FMD), using a 0.05 level of significance for between-group comparisons using a mixed effect logistic regression model. For this model, the dependent variable was the presence or absence of vesicular lesions, the explanatory variables were hours post inoculation and an indicator variable for challenge dose, and the individual animal identification was used as a random effect. Once the data from IOP-inoculated pigs were appropriately combined, the likelihood of the presence of vesicular lesions was estimated.

Similar to IOP-inoculated pigs, data from contact-exposed pigs from studies B and C (Study A did not contain data from contact pigs) were evaluated based on FMDV RNA dynamics in serum and OPF. The only fundamental difference between the GAMMs for the IOP-inoculated and contact-exposed was that we accounted for heteroskedasticity by weighting by the inverse variance within each of the pig groups in order to meet with the model assumption of constant variance. Data from Study C was evaluated first to determine differences between the 7 contact-exposed groups that were sequentially exposed to IOP-inoculated pigs through 8 h time slots from 8 to 56 hpi (23). The data from Study B containing 4 contact-exposed pigs that had been exposed to donor pigs from 36 to 60 hpi was then added to the Study C dataset. The exposure period of the contact-exposed pigs of Study B data matched the time of exposure of groups 4 through 7 from study C (32–56 hpi), but contributed unique data point measurements at 2, 4, 12, 15, and 18 hpi, allowing for better understanding of earlier dynamics (Table 1). The levels of FMDV RNA in serum or OPF were the dependent variables, while time (hours) and indicator variables based on exposure interval (in hpi) were the explanatory variables, and the individual animal identification was used as a random effect.

Using the combined datasets and final categories for contact-exposed pigs, two distinct GAMMs were fitted to the data. The levels of FMDV RNA in serum or OPF were the dependent variables, and (a smooth function of) time (hours) and category were the fixed explanatory variables, with individual animal identification used as a random effect to account for repeated measurements on the same animals.

Estimation of time to FMDV RNA detection in serum and OPF, as well as categorization based on the presence of vesicular lesions was performed using the same approach as described for the IOP-inoculated exposed pigs. However, for contact pigs, the explanatory variables were hours post exposure and category.

#### Estimating exposure dose in defined categories of IOP-inoculated and contact-exposed pigs

In order to investigate whether pigs with different infection dynamics had been exposed to different quantities of FMDV, the exposure dose of defined categories of contact-exposed and IOP-inoculated pigs were estimated and compared. Data from the three studies were included in this analysis as shown in Table 1. For this study, the estimated exposure dose was defined as the fitted mean concentration of FMDV RNA, expressed as log_10_ GCN/ml, detected in OPF samples of contact-exposed pigs at 8 hpe; or in OPF of IOP-inoculated pigs at 8 hpi (23). The infectious dose equivalents of all GCN values from OPF may be converted by the equation (Table S1):

$$\begin{array}{l} \text{TCI}{\text{D}}_{50}\;\left(\text{OPF}\right)=-3.63+\left(1.04\;*\text{ OPF }\log_{10}\text{ GCN}/\text{ml}\right) \end{array}$$

For IOP-inoculated pigs, 8 hpi represented the time at which residual inoculum was cleared and subsequently replaced by early de-novo replication of FMDV at the primary site of infection within the oropharynx. For the majority of contact-exposed pigs, 8 hpe corresponded to the end of the contact exposure period and therefore represented a point of cumulative viral exposure for these animals. This approach for estimating exposure dose, allowed us to determine the quantity of virus effectively received by each pig. For IOP-inoculated pigs, the estimated exposure dose at 8 hpi was distinct from the inoculated dose that was delivered at 0 hpi. For contact-exposed pigs, the estimated exposure dose at 8 hpe differed from the estimated virus shedding by the donor pigs at the corresponding time point. Estimating the effective exposure dose for both IOP inoculated and contact-exposed pigs allowed for comparable estimates between these two cohorts.

The estimation of the exposure dose in IOP-inoculated and contact-exposed pigs was done by fitting GAMMs as described above, using indicator variables for categories as explanatory variables in addition to the thin plate smooth term for time. The exposure dose for each of the categories of contact-exposed pigs and IOP-inoculated pigs was the fitted FMDV RNA estimate in OPF at 8 hpe or hpi, respectively. Model outputs are referred to as “estimated” or “fitted” values to distinguish from direct measurements.

The relationship between the amount of virus shed by donor pigs (FMD RNA in OPF at 8 h increments post-inoculation) and the amount of virus present in OPF of contact pigs (FMD RNA in OPF at 8 hpe) was examined using the data from Study C (23). A GAMM was fitted in which the FMDV RNA in OPF of each of the contact-exposed pigs measured at 8 hpe was used as the dependent variable. A thin plate smooth term for the mean of FMDV RNA in OPF of the donors at the end of each contact period was used as an explanatory variable. The effective degree of freedom (edf), F statistic, and a level of significance of < 0.001 were used to assess the relationship. If edf = 1, it indicates that the relation is linear, whereas if edf > 1 it is non-linear.

## Results

### Categorizing IOP-inoculated and contact-exposed pigs based on FMDV infection dynamics

Based on the GAMM results, there were no significant differences (*p* > 0.05) between the infection dynamics of the two IOP-inoculated dose-exposed groups [low (10 PHID_50_) and high (100 PHID_50_)] (data not shown). Therefore, all IOP inoculated pigs were treated as one category for subsequent analyses.

The eight contact–exposed groups of pigs from studies B and C were allocated into three categories based upon distinct infection dynamics, as determined by the GAMM. The categories were defined as: Category I (no clinical disease-low dose) included contact-exposed pigs from groups 1 and 2 from study C, Category II (delayed onset of clinical disease-mid dose) included contact-exposed pigs from group 3 from study C, and Category III (rapid onset of clinical disease-high dose) included contact-exposed pigs from groups 4–7 from study C and 4 contact-exposed pigs from study B (*p* > 0.05).

### FMDV infection dynamics of IOP-inoculated pigs

**Figure 1:**
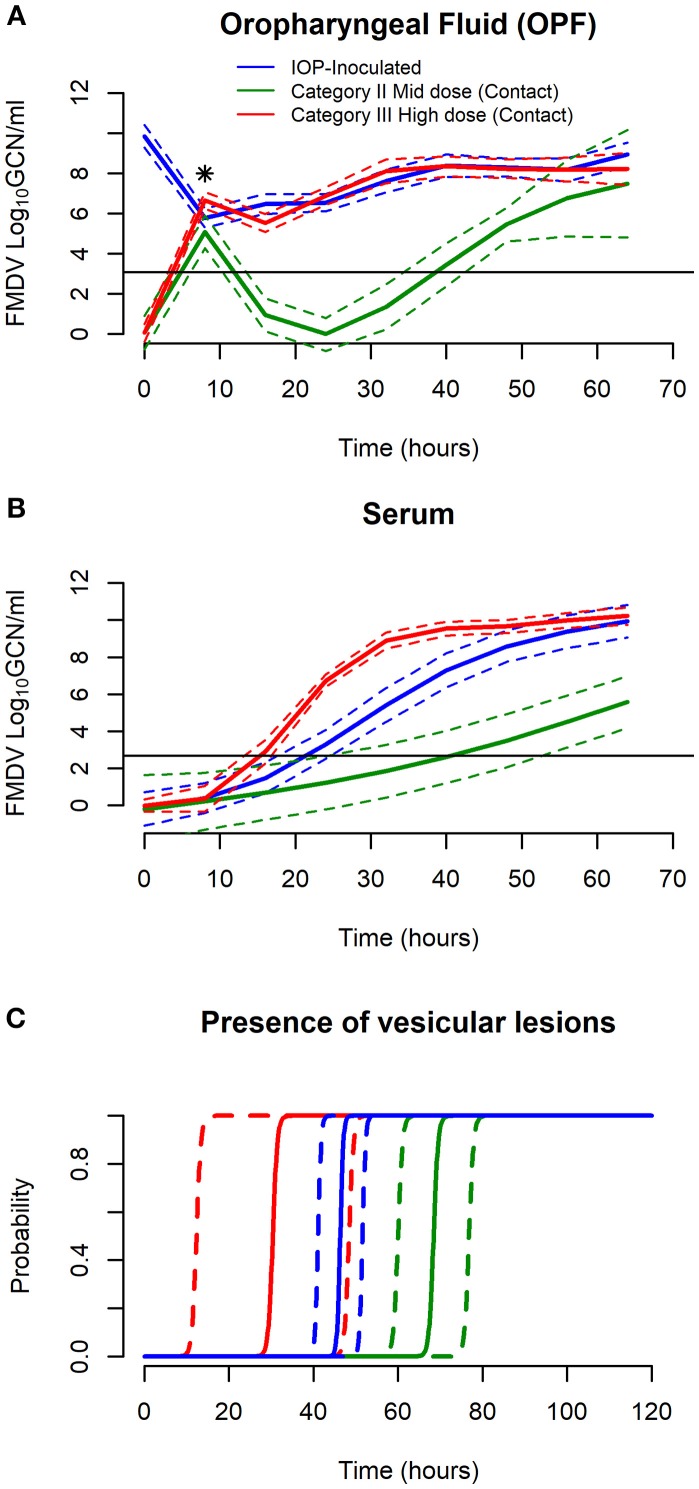
Comparison of infection dynamics between IOP-inoculated and Categories II and III of the direct contact-exposed pigs. **(A)** Estimated FMDV RNA dynamics in oropharyngeal fluid (OPF) [using Generalized Additive Mixed Models (GAMM)] are compared between contact categories (Categories II & III) and IOP-inoculated pigs. The black asterisk indicates the estimated effective exposure dose at 8 h post inoculation or exposure. The black horizontal line is the assay detection limit (3.08 log_10_ GCN/ml) expressed as log_10_ genome copy numbers (GCN)/ml. **(B)** Estimated FMDV RNA dynamics in serum (using GAMM) are compared between contact categories (Categories II & III) and IOP-inoculated pigs. The black horizontal line is the assay detection limit (2.68 log_10_ GCN/ml) expressed as log_10_ genome copy numbers (GCN)/ml. **(C)** Estimated probability of presence of vesicular lesions are compared between contact categories (Categories II & III) and IOP-inoculated pigs (using a mixed effect logistic regression model). For **(A–C)** figures, solid lines represent the fitted mean, while dashed lines represent 95% confidence intervals.

In IOP-inoculated pigs, quantities of FMDV RNA in OPF samples exceeded the lower limit of detection (3.08 log_10_ GCN/ml) throughout the entire study period (Table 2; Figure 1A). The estimated inoculated quantity of FMDV RNA (sampled immediately after inoculation) was 9.78 log_10_ GCN/ml (95% CI 9.23, 10.33; Figure 1). However, residual inoculum was cleared by 8 hpi as demonstrated by estimated FMDV RNA detection of 5.77 log_10_ GCN/ml (95% CI 5.30, 6.24; Figure 1, Table 2), corresponding to an estimated infectious dose of 10^2.36^ TCID_50_ (Table 3). FMDV detection in OPF increased steadily from this point until the end of the experiment representing *de-novo* viral replication. The maximum estimated de-novo viral replication of FMDV RNA quantity in OPF was 8.95 log_10_ GCN/ml (95% CI 8.37, 9.54) at 64 hpi (Table 2, Figure 1A).

**Table 2:** FMDV RNA detection in oropharyngeal fluid (OPF) of IOP-inoculated pigs as estimated by Generalized Additive Mixed Models (GAMM) fitted values.

	**Hours**	**Mean**	**95% CI**
	**(HPI)**	**(Log**_10_ **GCN/ml)**	
	8	5.77	5.30, 6.24
	12	5.92	5.45, 6.39
Latent	16	6.47	5.97, 6.96
	18	6.56	6.09, 7.02
^*^	24	6.55	6.11, 6.98
	32	7.63	7.06, 8.19
	40	8.39	7.83, 8.96
Infectious	48	8.29	7.84, 8.75
	56	8.18	7.61, 8.75
	64	8.95	8.37, 9.54

* *The dashed line represents the transition from latent to Infectious phase, which occurred between 24 and 32 HPI based upon experimental detection of transmission*.

**Table 3:** Estimated infectious dose threshold, and infective exposure dose between IOP-inoculated and contact-exposed pigs.

	**Estimated exposure dose**	

**Animal category**	**FMDV Genome copies^#^ (log_10_ GCN/ml)**	**Infectious dose equivalent^¥^ (log_10_ TCID_50_/ml)**
	**(mean [95% CI: Low, High])**	
IOP-Inoculated	5.77 [5.30, 6.24]	2.36 [1.88, 2.86]
Category I	N/A^*^	N/A^*^
Category II	5.07 [4.25, 5.89]	1.64 [0.79, 2.50]
Category III	6.67 [6.25, 7.08]	3.31 [2.87, 3.73]
	**Threshold of infectiousness**	
Donors	6.55 [6.11, 6.98]	10^∧^3.18 [2.72, 3.63]

* *There was no detection of FMDV in any samples from Category I (low dose) pigs*.

# *Estimated log_10_ GCN/ml, output from Generalized Additive Mixed Models*.

¥ *Infectious dose equivalent based on linear regression model (Table S1, Supplementary Material)*.

Detection of viremia (FMDV RNA in serum) was estimated to begin at 22 hpi (95% CI 18, 25) based upon a lower limit of detection of 2.68 log_10_ GCN/ml (Table 4; Figure 1B). From the earliest estimated detection in serum, FMDV RNA quantity continued to increase until the maximum estimated detection of 9.93 log_10_ GCN/ml (95% CI 9.05, 10.81) at 64 hpi (Figure 1B; Table 4). The estimated time of detection of the presence of vesicular lesions had a mean of 48 hpi (95% CI: 43, 54) (Figure 1C; Table 5). The mixed effects logistic regression model estimated a low probability (< 0.01) of presence of vesicular lesions in an IOP-inoculated pig as early as 24 hpi with probability increasing until 54 hpi. Specifically, it was estimated that at 48 hpi the probability of a pig having vesicular lesions was >0.99 (Figure 1C).

**Table 4:** FMDV RNA detection in serum of IOP-inoculated pigs as estimated by Generalized Additive Mixed Models (GAMM) fitted values.

	**Hours**	**Mean^$^**	**95% CI**

	**(HPI)**	**(Log_10_ GCN/ml)**	
	0	−0.20^*^	−1.10, 0.70
	8	0.39^*^	−0.41, 1.18
Pre-viremia	12	0.84^*^	0.01, 1.67
	16	1.47^*^	0.65, 2.30
#	18	1.87^*^	1.06, 2.68
	24	3.29	2.50, 4.07
	32	5.41	4.50, 6.32
Viremia	40	7.27	6.35, 8.20
	48	8.58	7.74, 9.42
	56	9.37	8.48, 10.26
	64	9.93	9.05, 10.81

* *FMDV was never experimentally detected in serum of these pigs prior to 24 HPI despite this modeled output*.

# *The dashed line represents the transition from pre-viremia to viremia, which occurred at 24 HPI based upon experimental detection*.

$ *FMDV RNA limit of detection in serum is 2.68 GCN mL*.

**Table 5:** Comparison of the estimated time of detection of presence of vesicular lesions using a mixed effect logistic regression model.

		**Time of detection (hours)**
Categories of Contact-Exposed Pigs	Category I (low dose)	N/A^*^
	Category II (mid dose)	72^#^ (66, 84)
	Category III (high dose)	36 (18, 54)
	IOP-Inoculated	48 (42, 54)

* *Category I (low dose) pigs never had detection of FMDV in OPF or serum despite having been exposed to donor pigs while they were shedding FMDV*.

# *Data reported as estimated hours (95% CI: Low, High)*.

### FMDV infection dynamics of contact-exposed pigs

The Category I pigs were not incorporated into GAMM analyses as none of the pigs became infected with FMDV despite exposure to donors with detectable FMDV RNA shedding. Confirmed lack of transmission to these groups was based upon lack of detection of FMDV RNA in serum and OPF samples, absence of vesicular lesions, and lack of seroconversion (23). For contact-exposed pigs of Categories II and III, early dynamics of FMDV RNA in serum and OPF, and the presence of vesicular lesions varied with respect to the estimated exposure dose. Category II pigs had a modeled delay of 36 (95%CI 32, 40) hours in detection of FMDV RNA in OPF samples compared to Category III pigs (Table 6). Similarly, mean time to detection of FMDV RNA in serum was 25 (95% CI 10, 36) hours delayed for Category II compared to Category III (Table 6). Comparison of the time to detection of FMDV RNA in OPF and serum indicated that FMDV RNA was detected earlier in OPF than in serum, in Category II and III. Also, compared to OPF, serum had higher variation of time to detection (see 95%CI of Table 6).

**Table 6:** Time of detection of FMDV RNA in oropharyngeal fluid (OPF) and serum from contact-exposed pigs as estimated by Generalized Additive Mixed Models (GAMM) fitted values.

		**Time of detection (Hours post exposure)**	

		**Oropharyngeal Fluid**	**Serum**
Categories of Contact-Exposed Pigs	Category I (low dose)	N/A^*^	N/A
	Category II (mid dose)	38.68^$^^,^^#^ (34.4, 42.46)	40.87 (24.41, 52.98)
	Category III (high dose)	2.52 (2.24, 2.94)	15.63 (13.9, 16.85)

* *Category I (low dose) pigs never had detection of FMDV in OPF or serum despite having been exposed to donor pigs while they were shedding FMDV*.

$ *Category II (mid dose exposed) contact group had OPF values above the limit of detection at 3 time points. However, based declining FMDV RNA detection at the 2 early time points [3.01 hpe (95% CI 2.17, 3.99) and 12.40 hpe (95% CI: 11.28, 13.66)], these detection events were interpreted to represent detection of shed virus from the donors, rather than virus replication and shedding by the contact pigs. Detection of FMDV RNA in the mid dose exposed group of pigs constantly above the limit of detection was in average at 38.68 hpe*.

# *Data reported as estimated hours post exposure (95% CI: Low, High)*.

The probability of the presence of vesicular lesions over time was influenced by the exposure dose of the pigs (*p* < 0.05) as shown by the differences between categories. For example, Category II pigs had < 0.01 probability of lesions at 48 hpe and >0.99 probability of having lesions at 72 hpe (Figure 1C, Table 5). In contrast, Category III pigs had probability of lesions at 48 hpe of nearly 1.0 with all pigs likely to have lesions by 72 hpe (Figure 1C, Table 5).

### Comparison of FMDV infection dynamics between IOP-inoculated and contact-exposed pigs

Comparison of infection dynamics between IOP-inoculated and contact-exposed pigs demonstrated that the infection dynamics of IOP-inoculated pigs was most similar to contact-exposed Category III pigs. Specifically, the mean time to detection of FMDV RNA in serum for IOP-inoculated pigs was 22 hpi, as compared to Category III contact-exposed pigs at 15.6 hpe and Category II at 40.8 hpe (Table 6). Similarly, the probability of presence of vesicular lesions in inoculated pigs was most similar to Category III pigs at the critical time points of 48 and 72 hpe (Figure 1C). Category III pigs progressed slightly faster than IOP-inoculated pigs in both lesion progression and viremia, whereas there was no distinguishable difference in the detection of FMDV in OPF (Figure 1).

### Estimated exposure doses for IOP-inoculated and contact exposed pigs

The quantity of FMDV RNA estimated in OPF samples of both IOP-inoculated and contact-exposed pigs at 8 h post inoculation or exposure was used as the proxy for the exposure dose. The rationale for using this time point was that for IOP-inoculated pigs the excess inoculum had been eliminated, and the contact-exposed pigs had accumulated their full exposure based on the experimental design.

**Figure 2:**
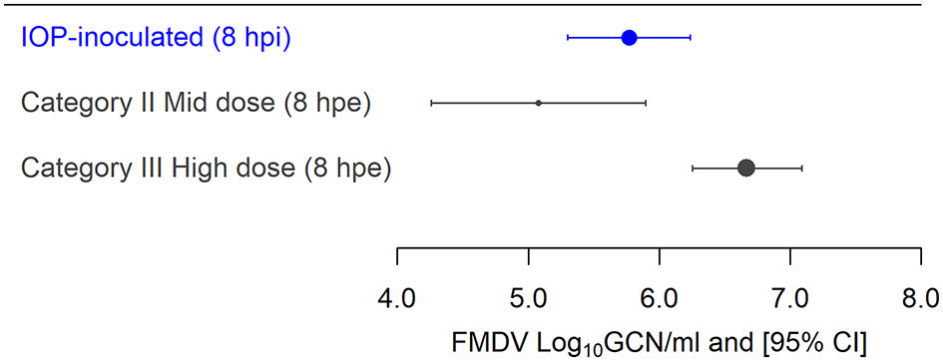
Estimated effective exposure dose of IOP-inoculated and contact-exposed pigs (Generalized Additive Mixed Models (GAMM) fitted values). Fitted mean and 95% Confidence Interval (CI) of FMDV RNA in the oropharyngeal fluid (OPF) of both IOP-inoculated pigs at 8 h post inoculation and direct contact exposed pigs at 8 h post exposure, expressed as log_10_ genome copy numbers (GCN)/ml. The size of the dots represents the number of animals in each category. Exposure dose could not be estimated for Category I pigs due to lack of detection of FMDV in OPF. Comparison of estimated doses of each category of contact-exposed pig and IOP-inoculated pigs.

For IOP-inoculated pigs, the estimated exposure dose at 8 hpi was 5.77 log_10_ GCN/ml (95% CI 5.30, 6.24), corresponding to an estimated infectivity of 10^2.36^ TCID_50_ (Table 3, Figure 2). The Category I contact-exposed pigs were exposed to the donors from 8 to 24 hpi, but did not become infected. Therefore, lack of detection of FMDV RNA in OPF at 8 hpe of Category I precluded the estimation of their exposure dose. The estimated exposure doses at 8 hpe for the two subsequent contact categories were: 5.07 log_10_ GCN/ml (95% CI 4.25, 5.89; 10^1.64^ TCID_50_) for Category II pigs and 6.67 log_10_ GCN/ml (95% CI 6.25, 7.08; 10^3.31^ TCID_50_) for Category III pigs (Table 3; Figure 2).

Furthermore, the minimum estimated exposure dose at which contact-exposed pigs became infected was 5.07 log_10_ GCN/ml (95% CI 4.25, 5.89; 10^1.64^ TCID_50_), corresponding to the minimum FMDV exposure of the Category II pigs. However, the minimum estimated shedding amount at which donor pigs infected contact pigs was 6.55 log_10_ GCN/ml (95% CI 6.11, 6.98; 10^3.18^ TCID_50_) (Table 2).

**Figure 3:**
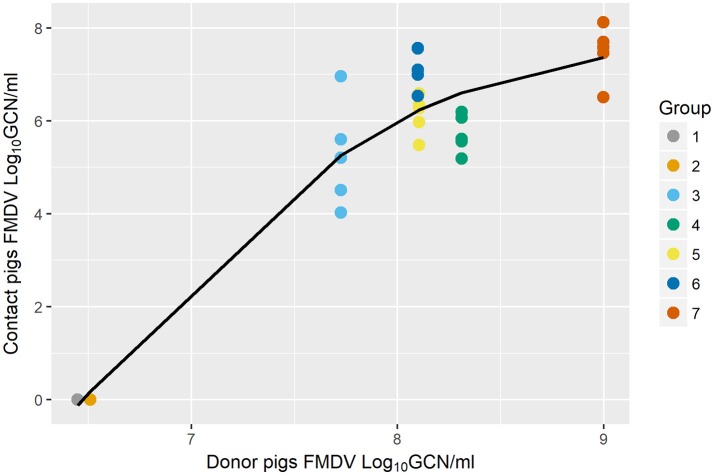
Scatter plot of the association between FMDV shedding of donors and the exposure dose of contact pigs [using Generalized Additive Mixed Model (GAMM)]. Quantitated FMDV RNA in the oropharyngeal fluid (OPF) measured at the end of the contact period in donor pigs explained the quantitated FMDV RNA in OPF of each of the sequentially direct contact-exposed groups (from Study C), measured at 8 h post exposure (*p* < 0.001). Colored circles represent raw data and the solid fitted line shows a non-linear relationship (*p* < 0.001).

The relationship between the shedding of FMDV RNA from donors and the quantitated exposure dose of contact groups was nonlinear, (edf = 1.968, *F* = 295.6, *p* < 0.001) but positively associated (Figure 3). The nonlinear relationship appears to be logarithmic over the values of donor shedding observed in the dataset in that it increases over a window and then levels off. The significance of the smooth term indicated that the exposure dose received by contact-exposed pigs was significantly explained by the quantitated shedding of FMDV RNA in OPF of donors (*p* < 0.001).

## Discussion

It is well-established that the inoculated dose of FMDV (8, 20, 32, 33) or the duration of contact exposure (18) may affect the infection dynamics of FMD in pigs, including the duration of the incubation (pre-clinical) period. Since detection of FMDV incursions depends, to a large extent, on the detection of clinical signs in infected animals, the effective virus dose to which animals have been exposed may potentially alter the time to detection of a new outbreak. However, no previous work has determined the effective exposure doses in contact-exposed pigs or quantitatively defined the manner in which infection dynamics are affected by different exposure doses. In order to investigate the effect of the dose of exposure on the early infection dynamics in FMDV-infected pigs, we analyzed data from experimental studies that were originally designed to characterize infection dynamics and transmission of FMDV A_24_ Cruzeiro in pigs infected by direct contact exposure or by simulated-natural intra oropharyngeal (IOP) inoculation.

For contact exposed animals, the Category I pigs, which had been exposed to donors from 8 to 24 hpi, were not infected despite consistent detection of shedding of low quantities of FMDV from the donors. The estimated range of FMDV infectious dose shedding in OPF over this time period was 10^2.1^–10^3.1^ TCID_50_/ml. This finding demonstrates that simply detecting FMDV shedding in pigs is not sufficient to define infectiousness, as has been done in previous meta-analyses to parameterize the latent and infectious periods of pigs for transmission models (34, 35). Furthermore, this finding demonstrates that transmission may be unlikely to occur unless a “threshold dose” is achieved. The threshold for transmission may be related to the instantaneous level of virus shedding from infected donor(s) or a cumulative duration of exposure dose over a period of contact. This finding is partially supported by Pacheco, Tucker (18), where the authors demonstrated that transmission efficacy varied based upon the duration of the exposure time, and the exposure time required for transmission differed with respect to different strains of FMDV.

For the Category II and III contact-exposed pigs, infection dynamics, including initial detection of FMDV shedding, onset of viremia, and occurrence of vesicular lesions were compared based upon the estimated FMDV exposure during contact. Category II pigs which were exposed to an estimated moderate exposure dose had delayed progression of disease relative to Category III pigs, which had a more rapid progression of FMD. Category II pigs were commingled with donors during the late incubation (pre-clinical) phase of infection, while Category III pigs commingled with donors during the onset of clinical signs and through the first 24 h of the clinical phase. This further supports the occurrence of pre-clinical infectiousness in pigs (23).

This approach also provides insight into the IOP inoculation system. The estimated dose of exposure for IOP-inoculated pigs was higher than the estimated minimum infectious exposure dose of contact pigs of Category II, but lower than the estimated exposure dose of contact pigs of Category III. Overall, this suggests that the IOP inoculation system delivers a dose of FMDV that is intermediate to the high and low boundaries of natural infection. This would suggest that the experimental studies based on IOP inoculation are likely to produce an average progression of FMD, while this analysis provides some estimate of the variability which can be seen in the timing of the disease process based on varying exposure doses that may be experienced naturally.

In addition to estimating the exposure dose of the contact-exposed pigs, we also estimated the FMDV shedding from the donors at the corresponding time points. The minimum estimated FMDV shedding in OPF at which donor pigs were capable of infecting contacts was estimated to range from 6.11 to 6.98 log_10_ GCN/ml (95% CI) with an average of 6.55 log_10_ GCN/ml, corresponding to an estimated infectious dose of 10^3.18^ TCID_50_. This threshold appears to coincide with the transition from latent to infectious. It is worth noting that the estimated amount of virus shed by donors before 24 hpi was only slightly lower than the quantities of FMDV RNA shedding at which donor pigs became infectious. The virus shedding threshold may have been reached earlier within the 8 h window of exposure, and additional measurements of virus shedding at more frequent time points could further refine the estimated shedding amount that results in transmission. Other previously reported measurements of shedding of FMDV, serotype O strain UKG2001, obtained from nasal swabs of needle-inoculated (intradermal or subcutaneous heel-pad) pigs that were capable of infecting contacts were 4.40 FMDV log_10_ GCN/ml and 6.47 FMDV log_10_ GCN/ml (19). Although there is some consistency in these findings with the present analysis, the variability in reported thresholds suggests that comparisons across studies may be difficult to achieve, given confounding factors such as different viral strains, sampling techniques, and the specific assays used for detection. The discrepancy between the estimated amounts of FMDV shed from donor pigs and the exposure dose received by contact pigs (6.55 > 5.07, respectively) may be explained by a possible degradation of the virus in the environment and that not all FMDV RNA quantified directly in the OPF of donors is shed to the environment.

Recent pathogenesis studies have demonstrated that in pigs, epithelial crypts within the oropharyngeal tonsils are the sites of primary FMDV infection and the major source of FMDV shedding throughout the clinical phase of disease. This favors the use of OPF over nasal secretions for quantitating shedding in pigs (9, 17, 24). The biological mechanisms by which a higher exposure dose accelerates infection dynamics remain unknown. Hypothetically, a greater initial dose might lead to more abundant foci of primary infection which would similarly lead to more rapid establishment of viremia, which is necessary for dissemination and clinical disease. However, there are other possible explanations and such hypotheses would need to be tested in controlled experiments in containment laboratories. For example, Quan et al. (20) investigated the differences of the rates of increase of FMDV in the blood at varying exposure doses, but no differences were found that could be explained by the exposure dose. Furthermore, the current study demonstrated a maximum estimated dose threshold (6.67 log_10_ GCN/ml [95% CI 6.25, 7.08]) beyond which infection dynamics did not proceed any faster. Specifically, there were no statistical differences between the infection dynamics of the four distinct groups of contact pigs within Category III (*p* > 0.05), even though FMDV shedding by donors gradually increased throughout the time during which these groups were exposed. A similar conclusion resulted from a previous study exploring exposure intensity on the efficacy and speed of transmission of FMD (19).

Three quantitative outcomes indicated that infection dynamics differed between the distinct contact categories: time to detection of FMDV RNA in serum and OPF and the probability of the presence of vesicular lesions. These measurements are commonly used to model disease dynamics scenarios, and other studies have highlighted the need for mathematical models of epidemiologic spread to accurately account for dose, infectiousness, and intensity of contact (19). Our results suggest that detection of FMDV RNA is achieved earlier in OPF samples than in serum, both of which are detectable prior to the occurrence of vesicular lesions. Therefore, FMDV RNA detection in OPF may be considered as the earliest effective screening test in the design of surveillance strategies, at the individual or herd level. Furthermore, these data demonstrate the importance of using complementary screening techniques during outbreaks, since FMD detection by each test may vary over time at the individual animal level.

The current study investigated the FMDV exposure dose as a critical biological factor to understand variability in infection dynamics. However, other biological factors have been reported to affect the infection dynamics of FMDV, such as the virulence of distinct virus strains, inoculation route, exposure intensity, and vaccination (8, 17–19). Due to consistent experimental design, these factors are unlikely to have influenced the outcomes of this analysis. However, the current study includes various assumptions and limitations which may restrict the extent to which the output is applicable to all FMD scenarios. Specifically, results from this study are based on a single virus strain, FMDV strain A_24_ Cruzeiro. Extrapolating the estimated exposure dose and shedding quantities to other FMDV strains may not be appropriate, as it has been demonstrated previously that FMDV dynamics and transmission can be strain-specific (18, 27). In addition, the homogeneity in the breed and age of pigs used in the current studies may differ from practical conditions in the field. Sample size is small, specifically for inoculated pigs receiving a dose of 10 PHID_50_ compared to 100 PHID_50_, therefore statistically significant differences may not be conclusive. Our results do provide, however, information on a range of plausible exposure doses that pigs would face in field conditions, corresponding to asynchronous infection dynamics of FMD in an outbreak situation.

In conclusion, these results demonstrated the importance of the effect of variable exposure dose during contact challenge on the infection dynamics of FMDV in pigs, and identified a threshold dose consistent with the onset of infectiousness. These novel approaches provide a previously unavailable approach for dose-quantitation which may be retrospectively applied to contact-exposure experiments or field scenarios. In addition, results from this study provided valuable context around how the exposure dose of the IOP route of inoculation related to the exposure dose experienced by contact-exposed pigs. Future work will focus on utilizing this information to model infection dynamics in populations of domestic pigs, under simulated outbreak conditions.

## Author contributions

KM-T, AD, CS, and JA: conceptualization of the research; CS and JA: data collection and testing; KM-T, MB, and BB: formal analyses; KM-T, BB, MB, AD, LR, CS, and JA: contributions to writing, reviewing and editing.

### Conflict of interest statement

The authors declare that the research was conducted in the absence of any commercial or financial relationships that could be construed as a potential conflict of interest.

## References

[1] Carpenter TE O'Brien JM Hagerman AD McCarl BA. Epidemic and economic impacts of delayed detection of foot-and-mouth disease: a case study of a simulated outbreak in California. J Veter Diagnost Investig. (2011) 23:26–33. 10.1177/104063871102300104 21217024

[2] Giles J. Delays allowed foot-and-mouth epidemic to sweep across Britain. Nature (2001) 410:501. 10.1038/35069198 11279447

[3] Grubman MJ Baxt B. Foot-and-mouth disease. Clin Microbiol Rev. (2004) 17:465–93. 10.1128/CMR.17.2.465-493.2004 15084510 PMC387408

[4] World Organisation for Animal Health (OIE) World Animal Health Information Database (WAHID) (2016). Available online at: http://www.oie.int/wahis/public.php?page=home (Accessed November 17, 2016).

[5] Pomeroy LW Bansal S Tildesley M Moreno-Torres KI Moritz M Xiao N. Data-driven models of foot-and-mouth disease dynamics: a review. Transbound Emerg Dis. (2015) 64:716–28. 10.1111/tbed.12437 26576514 PMC5205574

[6] Gale SB Miller GY Eshelman CE Wells SJ. Epidemic simulation of a foot and mouth disease outbreak in Minnesota. Rev Sci et Tech. (2015) 34:895–905. 10.20506/rst.34.3.2404 27044160

[7] Arzt J Baxt B Grubman MJ Jackson T Juleff N Rhyan J. The pathogenesis of foot-and-mouth disease ii: viral pathways in swine, small ruminants, and wildlife; myotropism, chronic syndromes, and molecular virus-host interactions. Transbound Emerg Dis. (2011) 58:305–26. 10.1111/j.1865-1682.2011.01236.x 21672184

[8] Stenfeldt C Pacheco JM Rodriguez LL Arzt J. Infection dynamics of foot-and-mouth disease virus in pigs using two novel simulated-natural inoculation methods. Res Veter Sci. (2014) 96:396–405. 10.1016/j.rvsc.2014.01.009 24548596

[9] Fukai K Yamada M Morioka K Ohashi S Yoshida K Kitano R. Dose-dependent responses of pigs infected with foot-and-mouth disease virus O/JPN/2010 by the intranasal and intraoral routes. Arch Virol. (2015) 160:129–39. 10.1007/s00705-014-2239-4 25281431

[10] Sellers R Gloster J. Foot-and-mouth disease: a review of intranasal infection of cattle, sheep and pigs. Veter J. (2008) 177:159–68. 10.1016/j.tvjl.2007.03.009 17509917

[11] Alexandersen S Donaldson AI. Further studies to quantify the dose of natural aerosols of foot-and-mouth disease virus for pigs. Epidemiol Infect. (2002) 128:313–23. 10.1017/S0950268801006501 12002550 PMC2869825

[12] Donaldson AI Alexandersen S Sorensen JH Mikkelsen T. Relative risks of the uncontrollable (airborne) spread of FMD by different species. Vet Rec. (2001) 148:602–4. 10.1136/vr.148.19.602 11386448

[13] Stenfeldt C Eschbaumer M Pacheco JM Rekant SI Rodriguez LL Arzt J. Pathogenesis of Primary Foot-and-Mouth Disease Virus Infection in the Nasopharynx of Vaccinated and Non-Vaccinated Cattle. PLoS ONE (2015) 10:e0143666. 10.1371/journal.pone.0143666 26599543 PMC4658095

[14] Stenfeldt C Pacheco JM Singanallur NB Ferreira HC Vosloo W Rodriguez LL. Clinical and virological dynamics of a serotype O 2010 South East Asia lineage foot-and-mouth disease virus in sheep using natural and simulated natural inoculation and exposure systems. Vet Microbiol. (2015) 178:50–60. 10.1016/j.vetmic.2015.04.004 25937316

[15] Arzt J Pacheco JM Rodriguez LL. The early pathogenesis of foot-and-mouth disease in cattle after aerosol inoculation:identification of the nasopharynx as the primary site of infection. Veter Pathol. (2010) 47:1048–63. 10.1177/0300985810372509 20587691

[16] Donaldson AI Alexandersen S. Relative resistance of pigs to infection by natural aerosols of FMD virus. Vet Rec. (2001) 148:600–2. 10.1136/vr.148.19.600 11386447

[17] Stenfeldt C Diaz-San Segundo F de los Santos T Rodriguez LL Arzt J The pathogenesis of foot-and-mouth disease in pigs. Front Veter Sci. (2016) 3:41 10.3389/fvets.2016.00041 PMC4876306 27243028

[18] Pacheco JM Tucker M Hartwig E Bishop E Arzt J Rodriguez LL. Direct contact transmission of three different foot-and-mouth disease virus strains in swine demonstrates important strain-specific differences. Veter J. (2012) 193:456–63. 10.1016/j.tvjl.2012.01.012 22342891

[19] Quan M Murphy CM Zhang Z Durand S Esteves I Doel C. Influence of exposure intensity on the efficiency and speed of transmission of Foot-and-mouth disease. J Comp Pathol. (2009) 140:225–37. 10.1016/j.jcpa.2008.12.002 19215941

[20] Quan M Murphy CM Zhang Z Alexandersen S. Determinants of early foot-and-mouth disease virus dynamics in pigs. J Comp Pathol. (2004) 131:294–307. 10.1016/j.jcpa.2004.05.002 15511538

[21] Alexandersen S Oleksiewicz MB Donaldson AI. The early pathogenesis of foot-and-mouth disease in pigs infected by contact: a quantitative time-course study using TaqMan RT-PCR. J Gen Virol. (2001) 82 (Pt 4):747–55. 10.1099/0022-1317-82-4-747 11257178

[22] Howey R Quan M Savill NJ Matthews L Alexandersen S Woolhouse M. Effect of the initial dose of foot-and-mouth disease virus on the early viral dynamics within pigs. J R Soc Inter. (2009) 6:835–47. 10.1098/rsif.2008.0434 19019816 PMC2838353

[23] Stenfeldt C Pacheco JM Brito BP Moreno-Torres KI Branan MA Delgado AH. Transmission of foot-and-mouth disease virus during the incubation period in pigs. Front Veter Sci. (2016) 3:105. 10.3389/fvets.2016.00105. 27917386 PMC5116750

[24] Stenfeldt C Pacheco JM Rodriguez LL Arzt J. Early events in the pathogenesis of foot-and-mouth disease in pigs; identification of oropharyngeal tonsils as sites of primary and sustained viral replication. PLoS ONE (2014) 9:106859. 10.1371/journal.pone.0106859 25184288 PMC4153717

[25] Pacheco JM Arzt J Rodriguez LL. Early events in the pathogenesis of foot-and-mouth disease in cattle after controlled aerosol exposure. Veter J. (2010) 183:46–53. 10.1016/j.tvjl.2008.08.023 18930417

[26] LaRocco M Krug PW Kramer E Ahmed Z Pacheco JM Duque H. A continuous bovine kidney cell line constitutively expressing bovine alphavbeta6 integrin has increased susceptibility to foot-and-mouth disease virus. J Clin Microbiol. (2013) 51:1714–20. 10.1128/JCM.03370-12 23515553 PMC3716081

[27] Pacheco JM Mason PW. Evaluation of infectivity and transmission of different Asian foot-and-mouth disease viruses in swine. J Vet Sci. (2010) 11:133–42. 10.4142/jvs.2010.11.2.133 20458154 PMC2873813

[28] R Core Team. R: A Language Environment for Statistical Computing. Version 313. Vienna: R Foundation for Statistrical Computing (2015). Available online at: http://www.R-project.org

[29] RStudio RStudio: Integrated Development Environment for R, Version 099357. Boston, MA (2009-2014).

[30] Wood S Mixed GAM Computation Vehicle with GCV/AIC/REML Smoothness Estimation. R package version 1.8-16 ed (2011). Available online at: https://stat.ethz.ch/R-manual/R-devel/library/mgcv/html/mgcv-package.html

[31] Zuur AF Ieno EN Walker NJ Saveliev AA Smith GM Mixed Effects Models and Extensions in Ecology with R. New York, NY: Springer (2009).

[32] Gloster J Doel C Gubbins S Paton DJ. Foot-and-mouth disease: Measurements of aerosol emission from pigs as a function of virus strain and initial dose. Veter J. (2008) 177:374–80. 10.1016/j.tvjl.2007.06.014 17827041

[33] Alexandersen S Quan M Murphy C Knight J Zhang Z. Studies of quantitative parameters of virus excretion and transmission in pigs and cattle experimentally infected with foot-and-mouth disease virus. J Comp Pathol. (2003) 129:268–82. 10.1016/S0021-9975(03)00045-8 14554125

[34] Mardones F Perez A Sanchez J Alkhamis M Carpenter T. Parameterization of the duration of infection stages of serotype O foot-and-mouth disease virus: an analytical review and meta-analysis with application to simulation models. Vet Res. (2010) 41:45. 10.1051/vetres/2010017 20205988 PMC2850150

[35] Kinsley AC Patterson G VanderWaal KL Craft ME Perez AM. Parameter values for epidemiological models of foot-and-mouth disease in swine. Front Veter Sci. (2016) 3:44. 10.3389/fvets.2016.00044 27314002 PMC4887472

