# Supplementary material for: Foot-and-Mouth Disease Infection Dynamics in Contact-Exposed Pigs Are Determined by the Estimated Exposure Dose
Source: Front Vet Sci. 2018 Jul 20;5:167. doi: 10.3389/fvets.2018.00167 (PMC6062637; doi:10.3389/fvets.2018.00167)
Supplement: Supplementary file 1 [file Table_1.DOCX]

Supplementary Material

**Foot-and-Mouth Disease Infection Dynamics in Contact-Exposed Pigs are determined by the Estimated Exposure Dose**

^X^Karla I. Moreno-Torres^1,2,3^, ^X^Barbara P. Brito^1,2^, Matthew A. Branan^3^, Luis L. Rodriguez^1^, Amy H. Delgado^3^, Carolina Stenfeldt^1,4*^, Jonathan Arzt^1*^

^*^ Corresponding author: Jonathan Arzt, [Jonathan.Arzt@ars.usda.gov](mailto:Jonathan.Arzt@ars.usda.gov)

Carolina Stenfeldt: [Carolina.Stenfeldt@ars.usda.gov](mailto:Carolina.Stenfeldt@ars.usda.gov)

Table S.1. Estimated TCID_50_/ml

| **Genome copies (log_10_ GCN/ml)** | **Infectious dose equivalent**  **(log_10_ TCID_50_/ml)** | |  |
| --- | --- | --- | --- |
|  | **OPF** | **Serum** |  |
| 3.5 | 0.01 | 0.43 |  |
| 4 | 0.53 | 0.95 |  |
| 4.5 | 1.05 | 1.47 |  |
| 5 | 1.57 | 1.99 |  |
| 5.5 | 2.09 | 2.51 |  |
| 6 | 2.61 | 3.03 |  |
| 6.5 | 3.13 | 3.55 |  |
| 7 | 3.65 | 4.07 |  |
| 7.5 | 4.17 | 4.59 |  |
| 8 | 4.69 | 5.11 |  |
| 8.5 | 5.21 | 5.63 |  |
| 9 | 5.73 | 6.15 |  |
| 9.5 | 6.25 | 6.67 |  |
| 10 | 6.77 | 7.19 |  |
|  |  |  |  |
| The estimated regression function to predict the TCID_50_ values from estimated GCN is  ${TCID}_{50} (OPF)=-3.63+(1.04*\mathrm{OPF}$log_10_ GCN/ml)  ${TCID}_{50}(SERUM)=-3.21+(1.04*\mathrm{SERUM}$log_10_ GCN/ml) | | | |
